# A meta-analysis of N-acetylcysteine in contrast-induced nephrotoxicity: unsupervised clustering to resolve heterogeneity

**DOI:** 10.1186/1741-7015-5-32

**Published:** 2007-11-14

**Authors:** Denise A Gonzales, Kelly J Norsworthy, Steven J Kern, Steve Banks, Pamela C Sieving, Robert A Star, Charles Natanson, Robert L Danner

**Affiliations:** 1 Critical Care Medicine Department, Clinical Center, National Institutes of Health, Bethesda, MD, USA; 2 National Institutes of Health Library, National Institutes of Health, Bethesda, MD, USA; 3 Division of Kidney, Urologic, and Hematologic Diseases, National Institute of Diabetes and Digestive and Kidney Diseases, National Institutes of Health, Bethesda, MD, USA

## Abstract

**Background:**

Meta-analyses of N-acetylcysteine (NAC) for preventing contrast-induced nephrotoxicity (CIN) have led to disparate conclusions. Here we examine and attempt to resolve the heterogeneity evident among these trials.

**Methods:**

Two reviewers independently extracted and graded the data. Limiting studies to randomized, controlled trials with adequate outcome data yielded 22 reports with 2746 patients.

**Results:**

Significant heterogeneity was detected among these trials (*I*^2 ^= 37%; *p *= 0.04). Meta-regression analysis failed to identify significant sources of heterogeneity. A modified L'Abbé plot that substituted groupwise changes in serum creatinine for nephrotoxicity rates, followed by model-based, unsupervised clustering resolved trials into two distinct, significantly different (*p *< 0.0001) and homogeneous populations (*I*^2 ^= 0 and *p *> 0.5, for both). Cluster 1 studies (*n *= 18; 2445 patients) showed no benefit (relative risk (RR) = 0.87; 95% confidence interval (CI) 0.68–1.12, *p *= 0.28), while cluster 2 studies (*n *= 4; 301 patients) indicated that NAC was highly beneficial (RR = 0.15; 95% CI 0.07–0.33, *p *< 0.0001). Benefit in cluster 2 was unexpectedly associated with NAC-induced decreases in creatinine from baseline (*p *= 0.07). Cluster 2 studies were relatively early, small and of lower quality compared with cluster 1 studies (*p *= 0.01 for the three factors combined). Dialysis use across all studies (five control, eight treatment; *p *= 0.42) did not suggest that NAC is beneficial.

**Conclusion:**

This meta-analysis does not support the efficacy of NAC to prevent CIN.

## Background

Since its development, meta-analysis has become a powerful tool for informing clinical practice. Performed correctly, meta-analysis is superior to a purely narrative approach of summarizing medical research. As such, robust conclusions may sometimes be reached from serial, otherwise underpowered small studies [1,2]. Nonetheless, there are substantial limitations and pitfalls in meta-analysis. Publication bias, reliance on subjective summary results rather than individual patient data and the mishandling of important heterogeneity can all lead to erroneous conclusions [1-8]. This possibility is underscored by the occasional lack of concordance between meta-analyses and subsequent large randomized, controlled trials [3,9].

Over the past decade, the efficacy of N-acetylcysteine (NAC) for preventing contrast-induced nephrotoxicity (CIN) has been explored in more than 60 clinical studies [10-71], 12 meta-analyses [72-83] and two comprehensive analyses of published meta-analyses [84,85]. Of the meta-analyses, some declared that NAC is beneficial [72-78] while others determined that the data are inconclusive [79-83]. Significant heterogeneity was detected in all of the meta-analyses that specifically tested for it and meta-regression and other approaches have failed to resolve or pinpoint the cause of the heterogeneity. This much-studied example, where meta-analysis may have increased rather than decreased clinical ambiguity, provides an opportunity to better understand and dissect complex heterogeneity problems in meta-analysis.

We assembled a meta-analysis of NAC efficacy in preventing CIN. Like previous attempts, we encountered significant heterogeneity that was not explained using a comprehensive meta-regression approach. A modified L'Abbé plot [86] followed by the application of a model-based, unsupervised clustering algorithm [87] resolved the trials into two significantly different populations. Clinical practices aimed at preventing CIN are discussed and recommendations are made regarding future trials of NAC.

## Methods

This meta-analysis was completed in accordance with the Quality of Reporting of Meta-analyses (QUOROM) statement [2].

### Literature search

We searched MEDLINE (PubMed and Dialog), EMBASE, International Pharmaceutical Abstracts, Derwent Drug File, Adis R&D Insight, Adis Clinical Trials Insight, Biological Abstracts and CINAHL (OVID), the Web of Science and The Cochrane Library. Searches included: controlled vocabulary for acetylcysteine, contrast media/adverse, toxic and poisoning effects; free text for acetylcysteine and contrast; and MeSH terms acetylcysteine and contrast media. Retrieved records from the Cochrane CENTRAL file were re-checked in Web of Science to identify subsequent publications. Search dates were from the inception of the databases until September 30, 2004. Conference proceedings from the American Society of Nephrology, National Kidney Foundation, American Heart Association, American College of Cardiology, Society of Interventional Radiology, Radiologic Society of North America and International Society of Nephrology were also reviewed over the past five years. There were no restrictions on language or publication status. Over 450 citations and abstracts were screened by two authors to assemble a preliminary set of possibly relevant reports. New publications after September 30, 2004 were periodically monitored using the same search criteria up to March 1, 2007.

### Selection criteria

Studies were limited to prospective, randomized, controlled trials (PRCTs) investigating the efficacy of NAC in preventing CIN. Trials with confounded, non-concurrent or otherwise improperly constructed control groups were prospectively excluded from further analysis. Outcome data were solicited from the authors if not found in the publication. Trials that still lacked outcome data necessary for planned analyses were excluded.

### Quality assessment, data retrieval and clinical endpoints

Two of the authors evaluated each trial using the Jadad scoring device, under unmasked conditions [88]. Each PRCT included in the analysis scored at least 1 on the five-point scale, with higher scores indicating greater trial quality. Data were extracted independently into a standardized form. Results were compared and disagreements were resolved by discussion. The primary outcome measures were the development of CIN as defined in the studies [10-31] and change in creatinine (ΔCre). The occurrence of acute kidney injury requiring dialysis was recorded. When not reported in the publication, we contacted the authors for post-contrast dialysis information.

### Meta-analysis and heterogeneity testing

Treatment effects were quantified by relative risk (RR) using a random-effects model (Comprehensive Meta-Analysis, Biostat Inc, Englewood, NJ). Statistical heterogeneity was assessed by means of a Mantel-Haenszel derived Cochran's *Q *statistic and associated *I*^2 ^value. Cochran's *Q *is used to test the null hypothesis that all treatment effects are equivalent [89]. Calculated from the *Q*-statistic and degrees of freedom, *I*^2 ^represents the proportion of treatment effect variation owing to trial heterogeneity, rather than simple sampling error [4,89,90]. Statistical heterogeneity is present when this variation in results exceeds the amount expected from chance alone. The quantitative pooling of such studies may lead to erroneous conclusions [4].

### Publication bias and meta-regression analysis

Evidence of publication bias was formally tested using multiple methods including those of Begg and Mazumdar [6], Egger *et al*. [5] and Higgins and Thompson [4]. Standard meta-regressions of the effect size expressed as log RR were performed against trial factors including publication date, size and Jadad score. Well-known patient-related risk factors associated with increased rates of CIN were also evaluated by meta-regression including mean age, diabetes mellitus (%), gender (% female), mean contrast volume and mean baseline creatinine concentration [91-94]. Likewise, total NAC dose was examined for its relationship with outcome. A separate meta-regression examined the log odds of developing CIN in the treatment *versus *the control groups. This was used to detect whether NAC efficacy was affected by the rate of CIN in the control population [95,96]. All meta-regressions were weighted by the inverse variance of each study.

### Jackknife-*k *sensitivity analysis, modified L'Abbé plot and unsupervised clustering: detection of trial subpopulations

A sensitivity analysis for heterogeneity was completed by means of a jackknife-*k *[97] procedure in order to detect studies that contributed most to heterogeneity. A pre-specified *p*-value greater than 0.2 for Cochran's *Q *statistic and an *I*^2 ^of less than 10% indicated homogeneity. Every possible one-, two- and three-study combination was removed.

The method of L'Abbé *et al*. [86] was used to visualize heterogeneity in our set of trials. As originally described, the L'Abbé plot graphs the control group outcome rate along the *x*-axis and the treatment group outcome rate along the *y*-axis for each trial. To correct for differences in the definition of CIN across studies, we modified the L'Abbé plot by substituting ΔCre, a continuous variable, for the CIN rate. Compared with a standard L'Abbé plot (data not shown), the modified plot was similar, but was better at separating studies that were low and high contributors to heterogeneity.

We then analyzed our modified L'Abbé plot using an unsupervised, model-based clustering method that creates a best-fit Gaussian model and finds the number of clusters that maximize the Bayesian information criterion. All members of the data set are then classified using iterative expectation-maximization methods and group membership likelihoods are calculated [87]. The study and patient characteristics of each cluster were then compared using Wilcoxon rank sum tests. The decomposed Breslow-Day test was used to determine whether the identified clusters had significantly different treatment effects.

## Results

### Trial flow

**Figure 1:**
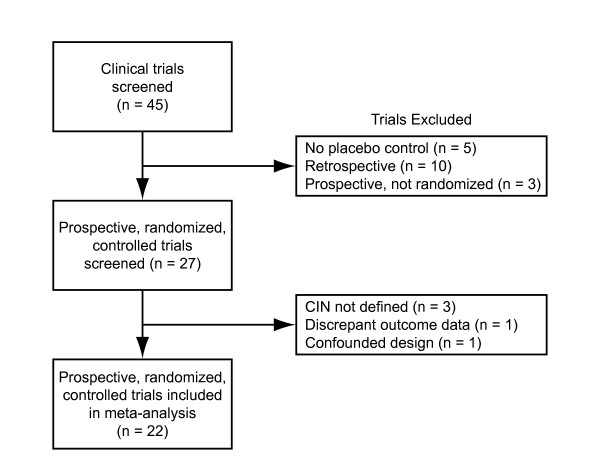
Study selection flow diagram.

The literature search identified 45 clinical studies investigating NAC to prevent CIN (Figure 1). Ten studies were retrospective [32-41]. Three studies were prospective but not randomized [42-44]. Five studies were removed owing to a lack of placebo controls [45-49]. Three studies were excluded because CIN was not clearly defined [50-52]. One abstract was excluded because discrepant outcome results reported in the abstract and a subsequent meta-analysis could not be resolved [53]. One study was removed owing to a confounded design, where treated patients received more fluid compared with controls[54].

Additional information required for analysis was requested from trial authors; when unsuccessful in the case of one abstract [18], data were extracted from other meta-analyses. We included the more complete, updated data from manuscripts that were published after our cut-off date [55-57] if these studies had been available in the form of abstracts [19-21] before September, 2004.

**Figure 2:**
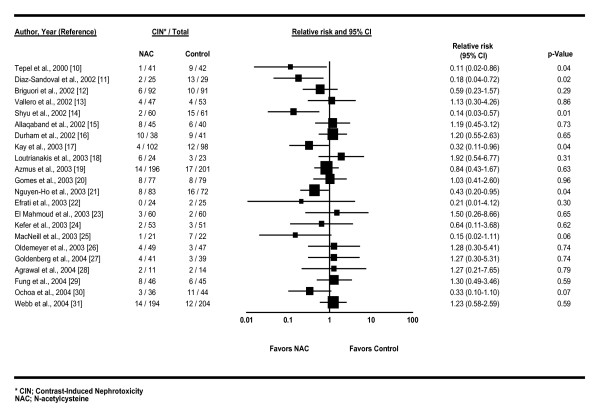
**Forest plot of twenty-two studies meeting inclusion criteria for meta-analysis**. Studies are ordered by date of publication. Lines represent 95% CIs. Box sizes represent the weight (by inverse variance) of each trial. Note a trend over time towards no effect. No summary statistic is shown owing to excessive heterogeneity.

Table 1 lists the characteristics of the 22 trials meeting our prospective selection criteria [10-31]. Figure 2 shows a forest plot ordered by time of publication, with RR and confidence intervals (CIs) of developing CIN if treated with NAC. A summary statistic is not shown owing to the significant heterogeneity (*I*^2 ^= 37%; *p *= 0.04) that precluded the pooling of these trials.

**Table 1:** Study Patient Characteristics

**Authors (reference)**	**Pub. Date**	**Average patient age (years)**	**Per cent Male**	**BSCr (mg/dl)**	**Diabetes (%)**	**Contrast volume (ml)**	**Jadad Score**	**End point (SCr rise)**	**Contrast procedure**	**NAC regimen**	**Hydration regimen**
Tepel *et al*. [10]	*07/00*	*65*	NA	*2.5*	*32.5*	*75*	*1*	*0.5 mg/dl 48 h*	*CT*	*600 mg tablet bid × 4*	*0.45% 1 ml/kg/h 12 h before, 12 h after*
Diaz-Sandoval *et al*. [11]	*02/02*	*73*	*80%*	*1.6*	*38.9*	*184*	*2*	*0.5 mg/dl or 25% 48 h*	*LHC*	*600 mg liquid in ginger ale bid × 4*	*0.45% 1 ml/kg/h 2–12 h before, 12 h after*
Briguori *et al*. [12]	07/02	64	86%	1.5	37.8	197	1	25% 48 h	LHC and/or PA and/or PCI	600 mg tablet bid × 4	0.45% 1 ml/kg/h 12 h before, 12 h after
Vallero *et al*. [13]	09/02	62	NA	1.0	23.0	205	1	0.5 mg/dl or 33% 48 h	LHC and/or PCI	600 mg tablet bid × 4	0.45% 1 ml/kg/h 1–2 h before, 24 h after
Shyu *et al*. [14]	*10/02*	*70*	*68%*	*2.8*	*63.5*	*117*	*1*	*0.5 mg/dl 48 h*	*LHC ± PCI*	*400 mg powder bid × 4*	*0.45% 1 ml/kg/h 12 h before, 12 h after*
Allaqaband *et al*. [15]	11/02	70	NA	2.1	48.3	122	3	0.5 mg/dl 48 h	LHC ± PCI or PA + PCI	600 mg liquid in cola bid × 4	0.45% 1 ml/kg/h 12 h before, 12 h after
Durham *et al*. [16]	12/02	71	66%	2.3	48.1	81	3	0.5 mg/dl 48 h	LHC	1200 mg liquid in orange juice bid × 2	0.45% 1 ml/kg/h ≤ 12 h before, ≤ 12 h after
Kay *et al*. [17]	02/03	69	62%	1.3	37.5	125	5	25% 48 h	LHC and/or PCI	600 mg tablet bid × 4	0.9% 1 ml/kg/h 12 h before, 6 h after
Loutrianakis *et al*. [18]	03/03	67	NA	1.9	36.0	147	1	0.5 mg/dl 120–168 h	LHC	600 mg bid × 4	0.45% 1 ml/kg/h
Azmus *et al*. [19]	07/03	67	59%	1.3	49.6	126	5	0.5 mg/dl or 25% 24–48 h	LHC or PCI	600 mg powder in water bid × 5	0.9% 1 L pre, 1 L post, or none
Gomes *et al*. [20]	10/03	65	59%	1.3	51.9	103	4	0.5 mg/dl 48 h	LHC or PCI	600 mg bid × 4	0.9% 1 ml/kg/h 12 h before, 12 h after
Nguyen-Ho *et al*. [21]	11/03	70	NA	1.4	67.5	347	4	25% 48–72 h	LHC or PCI	2000 mg liquid in juice bid × 2 or 3	0.45% 75 ml/h ≥ 24 h from enroll
Efrati *et al*. [22]	12/03	67	90%	1.5	52.9	140	2	25% 24–96 h	LHC	1000 mg liquid in cola bid × 4	0.45% 1 ml/kg/h
El Mahmoud *et al*. [23]	12/03	67	81%	1.9	30.0	177	2	25% 24–48 h	LHC	600 mg orally bid × 2	0.9% 1 ml/kg/h
Kefer *et al*. [24]	12/03	62	77%	1.1	12.5	199	1	0.5 mg/dl or 25% 24 h	LHC and/or PCI	1200 mg in 0.9% saline IV over 60 min, 12 h pre 0 h post	0.9% 1 ml/kg/h
MacNeill *et al*. [25]	*12/03*	*73*	*86%*	*1.9*	*46.5*	*110*	*4*	*25% 72 h*	*LHC ± PCI*	*600 mg liquid in juice/soda bid *×*5*	*0.45% 1 ml/kg/h 12 h or 2 ml/kg/h 4 h before, 75 ml/h 12 h after*
Oldemeyer *et al*. [26]	12/03	76	55%	1.6	44.9	131	2	0.5 mg/dl or 25% 48 h	LHC	1500 mg liquid in soda bid × 4	500 ml D5 20 ml/h 12 h before, 12 h after
Goldenberg *et al*. [27]	02/04	70	83%	2.0	43.9	116	5	0.5 mg/dl 48 h	LHC ± PCI	600 mg liquid in soda tid × 6	0.45% 1 ml/kg/h
Agrawal *et al*. [28]	04/04	63	68%	1.7	47.8	178	2	0.5 mg/dl or 25% 48 h	LHC and/or PCI	800/600/600 mg liquid in soda 12/2 h pre/6 h post	0.45% 1 ml/kg 12 h ± 250 ml bolus before, 12 h after
Fung *et al*. [29]	05/04	68	70%	2.3	52.8	128	3	0.5 mg/dl or 25% decrease in GFR 48 h	LHC or PCI ± PA	400 mg powder tid × 6	0.9% 100 ml/h 12 h before, 12 h after
Ochoa *et al*. [30]	06/04	71	43%	2.0	55.5	144	4	0.5 mg/dl or 25% 48 h	LHC and/or PCI	1000 mg liquid in diet cola bid × 2	0.9% 150 ml/h, ≥ 500 ml 12 h before, ≥ 1000 24 h after
Webb *et al*. [31]	09/04	70	NA	1.7	34.9	120	5	0.5 mg/dl 48–192 h	LHC or PCI ± PA	500 mg in D5NS IV for 15 min, 1 h pre	0.9% 200 ml before, 1.5 ml/kg/h 6 h or discharge (<6 h) after

SCr, serum creatinine; BSCr, baseline serum creatinine; CT, computed tomography; LHC, left heart catheterization; PCI, percutaneous coronary intervention; PA, peripheral angiography; Jadad score, measure of study design quality (0 is the weakest, 5 is the strongest); NAC, N-acetylcysteine; NA, not applicable; bid, twice daily; tid, three times daily; IV, intravenous; h, hour; D5NS, 5% dextrose plus normal saline; 0.9%, normal saline; 0.45%, half-normal saline.

### Publication bias and meta-regression analysis

**Figure 3:**
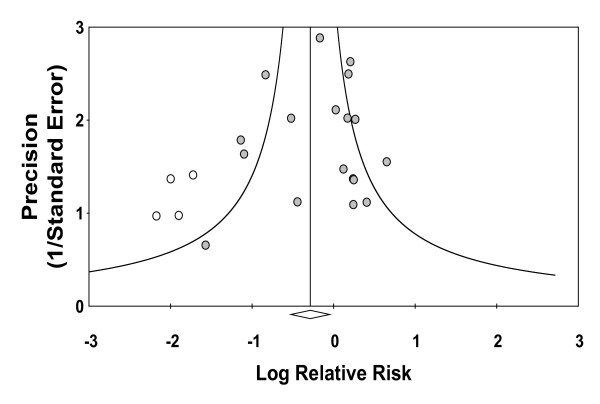
**Funnel plot of precision versus log RR**. Log RR of developing CIN is plotted versus precision for each of the 22 studies in this meta-analysis. Four studies later identified as contributing most to heterogeneity are noted with open circles and are seen to produce asymmetry in the plot. The summary log RR for all 22 studies is denoted by the open diamond.

Although non-significant (*p *≤ 0.11, but *p *> 0.05 when applying any one of the three methods used for analysis), a visual inspection of a funnel plot suggested publication bias with four studies [10,11,14,25] contributing most to the apparent asymmetry (shown with open circles on the left-hand side of Figure 3). An extensive meta-regression analysis of patient and study characteristics found no study-specific characteristic (publication date, size, quality as measured by Jadad score or total NAC dose) or patient-related characteristic (age, diabetes, gender, contrast volume, baseline creatinine or CIN event rate in the control group) that significantly co-varied with NAC efficacy (Table 2).

**Table 2:** Meta-regression of study and patient factors

	**Characteristic**	r*	***p*-value**
**Study-related**	Publication date (months after first)	0.36	0.1
	Study size (number of patients)	0.14	0.54
	Jadad score (1–5)	0.07	0.75
	Total NAC dose (mg)	-0.26	0.25
**Patient-related**	Age (years)	-0.13	0.56
	Baseline Creatinine (mg/dl)	-0.01	0.96
	Diabetes mellitus (%)	-0.23	0.31
	Female (%)	0.1	0.72
	Contrast volume (ml)	-0.27	0.24
	CIN event rate in control group (%)	0.21	0.35

*A negative correlation coefficient implies more benefit as the tested independent variable increases.

### Sensitivity analysis

**Figure 4:**
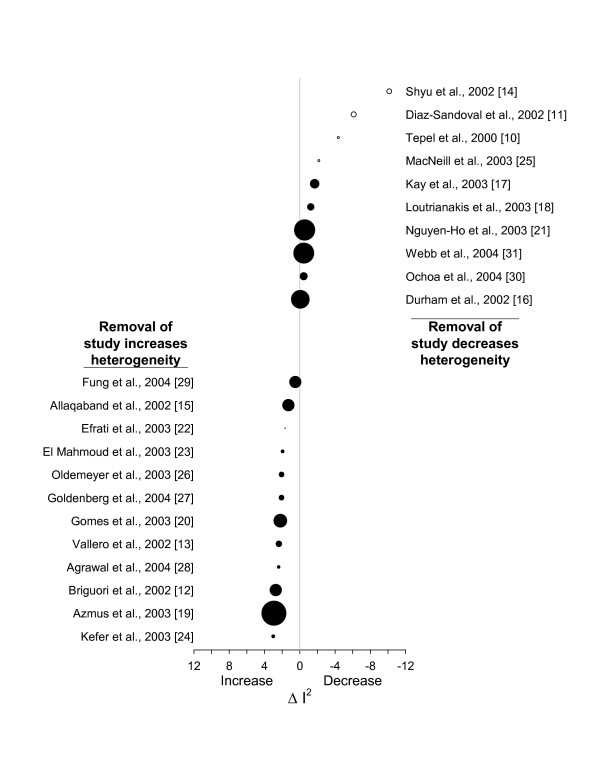
**Jackknife sensitivity analysis**. Studies are ordered from top to bottom by their effect on heterogeneity when removed one at a time from the set of 22 studies. Removing any of the 10 studies at the top of the plot decreases heterogeneity, while removing any of the 12 studies at the bottom of the plot increases heterogeneity. The four studies that individually contributed the most to heterogeneity are shown as open circles. Circle size is proportional to the inverse variance.

A jackknife-*k *sensitivity analysis [97] identified 10 studies that decreased heterogeneity when individually removed (right-hand side of Figure 4). Removal of any one of the remaining 12 studies increased heterogeneity (left-hand side of Figure 4). The four small studies [10,11,14,25] that individually contributed the most to heterogeneity are shown as open circles in Figure 4 (circle size is proportional to inverse variance). Removal of any single study or all possible two-study combinations failed to adequately resolve heterogeneity. In contrast, the removal of multiple three-study combinations (combinations [11,14,25][10,11,14][11,14,21] and [11,14,17]) reached our pre-defined target for homogeneity (after the removal of any one of the three-study groups above, *I*^2 ^≤ 9.5% and *p *≥ 0.34). These four three-study groups represent only 7.9%, 9.4%, 12.0% and 13.7% of the entire study population, respectively.

### Modified L'Abbé plot and unsupervised clustering analysis

**Figure 5:**
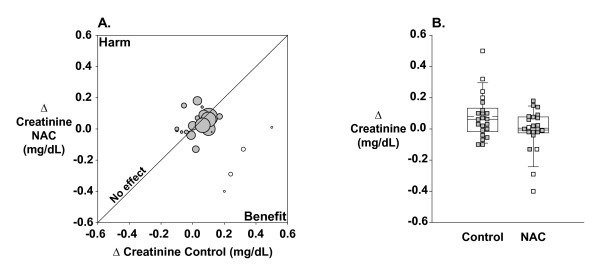
**Changes in creatinine across all trials**. **A**: Modified L'Abbé plot of change in creatinine from baseline to study endpoint in the control arm (*x*-axis) versus NAC treatment arm (*y*-axis) of each study. Studies are weighted by inverse variance (i.e. larger symbols represent larger studies with less variability). Open circles denote cluster 2 studies [10, 11, 14, 25]. **B**: Box plot of change in creatinine from baseline to study endpoint in the control arm and NAC treatment arm of each study. Boxes represent the 25th, 50th and 75th percentiles. Whiskers are 5th and 95th percentiles. Dashed lines show the mean of each group. Open squares denote cluster 2 studies.

A modified L'Abbé plot of creatinine change in controls versus creatinine change in NAC-treated subjects for all 22 studies is shown in Figure 5A. The no-effect line is plotted for reference. Most trials grouped together symmetrically around the no-effect line, with the exception of four very beneficial, relatively small studies [10,11,14,25]. These same four studies had caused the appearance of asymmetry in the funnel plot and were associated with heterogeneity by jackknife-*k *analysis. As suggested by the L'Abbé plot, a box plot (Figure 5B) of creatinine change clearly shows that these four studies have relatively large creatinine increases in control patients (*p *= 0.02; open boxes on the left-hand side) and relatively large creatinine decreases in NAC-treated patients (*p *= 0.07; open boxes on the right-hand side).

**Figure 6:**
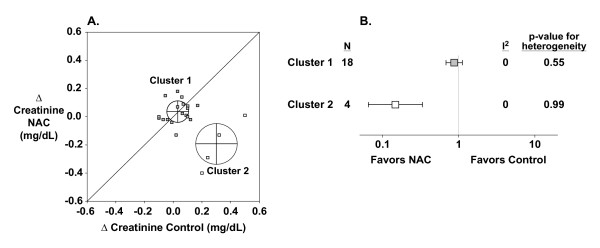
**Cluster analysis based on changes in creatinine**. **A**: Modified L'Abbé plot showing the results of model-based, unsupervised cluster analysis. Unlike Figure 5A, studies are unweighted for easier visualization. Cluster analysis (see the Methods section) applied to the 22 studies found two distinct populations of trials. Crosshairs and circles denote the mean ± SD of each cluster. **B**: Aggregate NAC treatment effect and heterogeneity analysis of each cluster. The entire group of 22 studies had unacceptable heterogeneity (*I*^2 ^= 37%; *p *= 0.04) making the summary point estimate unreliable (not shown). Cluster 1 (*n *= 18; 2445 patients) is homogeneous and shows no benefit (RR = 0.87; 95% CI 0.68–1.12, *p *= 0.28). Cluster 2 (*N *= 4; 301 patients) is also homogeneous and indicates that NAC is very beneficial (RR = 0.15; 95% CI 0.07–0.33, *p *< 0.0001).

Using a model-based, unsupervised clustering approach [87], our modified L'Abbé plot defined two different subpopulations of trials within the overall meta-analysis (Figure 6A). Dividing the 22 PRCTs based on their assignment to cluster 1 (18 studies, 89% of patients) [12,13,15-24,26-31] or cluster 2 (four studies, 11% of patients) [10,11,14,25], these two sets of trials were found to have significantly different treatment effects (*p *< 0.0001) and both were internally homogeneous (Figure 6B). Group membership likelihoods were greater than 90% for the 18 studies assigned to cluster 1 and greater than 99% for the four studies assigned to cluster 2. Cluster 1 studies (2445 patients) showed no benefit from NAC administration to prevent CIN (RR = 0.87; 95% CI 0.68–1.12, *p *= 0.28). Cluster 2 studies (301 patients) indicated a large benefit from NAC treatment (RR = 0.15; CI 0.07–0.33, *p *< 0.0001). The four highly beneficial trials in cluster 2 all employed oral NAC at low or moderate doses and in this regard were not different in design from some larger trials that grouped with cluster 1. Likewise, cluster 2 patients received iopromide, ioxilan or iopamidol, contrast agents which did not appear to explain the large apparent benefit of NAC in these studies. However, cluster 2 studies were published earlier, are smaller in size and of lower quality as measured by Jadad scores (Table 3; *p *= 0.01, three study characteristics combined). Notably, control subjects experienced more CIN in cluster 2 compared with cluster 1 trials (31% ± 10% versus 12% ± 6%; *p *= 0.03). These increased episodes of CIN in cluster 2 were not associated with any consistent pattern of patient-related characteristics that increase risk for CIN (Table3).

**Table 3:** Comparison of cluster 1 and cluster 2 studies (mean ± SD)

	**Characteristic**	**Cluster 1**	**Cluster 2**	***p*-value**^†^
**Study-related**	Publication date (months after first)	38 ± 8	22 ± 17	0.05
	Study size (number of patients)	136 ± 106	75 ± 35	0.23
	Jadad score (1–5)	2.9 ± 1.5	2.0 ± 1.4	0.24
	All three factors combined	34 ± 13	50 ± 9	0.01
**Patient-related**	Age (years)	68 ± 4	70 ± 3	0.24
	Baseline creatinine (mg/dl)	1.6 ± 0.4	2.2 ± 0.6	0.09
	Diabetes mellitus (%)	43 ± 13	45 ± 13	0.93
	Female (%)	31 ± 14	22 ± 9	0.31
	Contrast volume (ml)	158 ± 61	122 ± 46	0.11

† Wilcoxon rank sum test

### Power analysis

A power analysis was performed using the point estimate of the treatment effect in cluster1 trials (RR = 0.87) to provide the most conservative estimate of the size of a trial necessary to show a significant effect. A single PRCT comparing NAC treatment with control subjects, in a balanced design, would need to enroll 32 200 patients in order to have an 80% chance of showing a significant benefit of NAC to prevent CIN at the *p *< 0.05 level. This assumes that the diagnosis of CIN would be based on similar cut-off values for a change in creatinine [10-31].

### Dialysis events after contrast

The occurrence of dialysis was examined in the 22 trials meeting our inclusion criteria (*n *= 2746). A total of 13 patients received dialysis post-contrast (control *n *= 5, NAC-treated *n *= 8; *p *= 0.42) with no difference in the use of dialysis in cluster 1 (control *n *= 4, NAC-treated *n *= 8; *p *= 0.26) and cluster 2 (control *n *= 1, NAC-treated *n *= 0; *p *= 1.0) between the two treatment arms. NAC treatment showed no evidence of being protective using the clinical endpoint of dialysis events (RR = 1.42; CI 0.46–4.39, *p *= 0.54).

### Examination of new studies published after our cut-off date

From September 30, 2004 to March 1, 2007, 14 clinical trials of NAC in CIN were published [58-69]. Nine studies [58-64,70,71] met our prospective inclusion criteria. Like our meta-analysis of 22 PRCTs, these nine trials (1151 patients) had significant heterogeneity (*I*^2 ^= 56.0%; *p *= 0.03). When the nine studies were added to our meta-analysis, significant heterogeneity was again observed (*I*^2 ^= 40.9%; *p *= 0.01). Our model-based, unsupervised clustering approach showed that eight of these trials [58-63,70,71] grouped with cluster 1 with a probability of group membership of more than 94% for each trial. This updated cluster 1 (26 studies, 3268 patients) had low, non-significant heterogeneity (*I*^2 ^= 8.3%; *p *= 0.34) and showed no benefit of NAC for preventing CIN (RR = 0.90; 95% CI 0.72–1.12, *p *= 0.35). Cluster 1 and 2 treatment effects remained significantly different (*p *< 0.0001).

One study of both high- and low-dose intravenous NAC in patients with acute myocardial infarctions [64] did not group strongly with either cluster (probabilities of group membership: low-dose arm, 39% for cluster 1 and 61% for cluster 2; high-dose arm, 49% for cluster 1 and 51% for cluster 2). Based on these results, this study [64] was found to be an outlier (*p *< 0.05; Dixon test) [98] compared with other trials assigned to either cluster 1 or 2.

### Hemodialysis risk model

**Figure 7:**
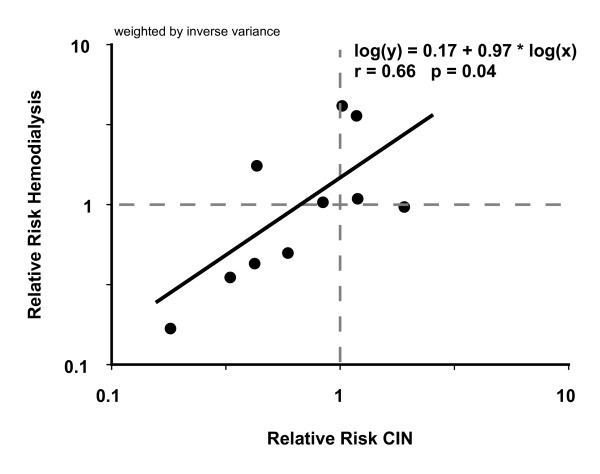
**Hemodialysis risk model**. Relative risk of developing CIN is plotted versus RR of needing hemodialysis, based on hemodialysis data available from nine studies. Axes are in logarithmic scale. The RR of CIN would have to be less than 0.67 in order for the RR of hemodialysis not to be on the side of harm (RR < 1).

We tested for a correlation between CIN and the clinically more rigorous outcome of dialysis. The correlation was weighted by the inverse variance of each study. Of the 22 trials in our meta-analysis and the nine more recent studies, hemodialysis events occurred in a total of nine trials [12,15,16,18-21,58,64]. Figure 7 shows that the RR of CIN, as defined in each trial, is positively correlated with the RR of requiring dialysis post-contrast (*r *= 0.66; *p *= 0.038). However, the regression equation is shifted upwards from the line of identity. For the RR of dialysis to be on the side of benefit (RR < 1.0), the RR of CIN would need to be substantially below one (RR < 0.67 for CIN). In fact, observing a RR of CIN this low in any future clinical trial is unlikely based on our cluster analysis, because it lies outside the 95% CI for cluster 1. A trial enrolling 32 200 patients, as described in the power analysis, would also have a moderate likelihood of showing a harmful effect of NAC on the need for post-contrast dialysis (RR = 1.29).

## Discussion

The limited ability of meta-analysis to address unexplained heterogeneity has been explored in a well-known data set that has been subjected to a large number of previous investigations. CIN is a common and important complication of diagnostic imaging that has a substantial impact on morbidity and mortality [91-94]. While hydration is clearly beneficial in preventing CIN [99,100], NAC has been investigated in many trials and subsequent meta-analyses with no consistent answer as to its efficacy. This meta-analysis of 22 studies, like previous meta-analyses [72-83], has demonstrated significant heterogeneity. The inconsistency across studies was systematically explored. Funnel plots [4-6] and a reiterative sensitivity analysis [97] both identified subsets of studies that appeared to be most strongly associated with this problem. However, a standard meta-regression approach [1,2,84] failed to identify a single study or patient-related characteristic that correlated with or fully explained variability in the NAC treatment effect. Ultimately, a modified L'Abbé plot [86] that substituted change in creatinine, a directly measured continuous endpoint, for CIN event rates, an all-or-nothing outcome that was variably defined across trials, indicated the possibility of distinct trial subpopulations within the overall results. Borrowing from our experience in functional genomics research, unsupervised, model-based clustering [87] was applied to demonstrate that the data set represented two homogeneous, significantly different trial populations. This novel approach allowed us to directly compare trials that populated each of the two dissimilar clusters and provided a reliable aggregate point estimate for performing a formal power analysis.

NAC prophylaxis for the prevention of CIN was first introduced in 2000 [10] and although definitive proof of efficacy has been elusive, the use of NAC prophylaxis has become widespread. NAC trials have mainly been conducted in stable patient populations with at least one risk factor for the development of CIN [10-68]. Small doses of NAC given orally have been the most frequently investigated regimen despite evidence that the drug is poorly absorbed and undergoes significant first-past metabolism [101]. Although vigorous hydration has been demonstrated as an effective preventive strategy [99], NAC trials have typically been conducted using no more than maintenance infusions (1 ml/kg/h) of half-normal or normal saline [10-31]. Whether the small, non-significant benefit of NAC in cluster 1 of our meta-analysis would persist if hydration were individually optimized is questionable. Importantly, a large PRCT of unselected patients undergoing elective coronary angiography found that normal compared with half-normal saline reduced the incidence of CIN almost threefold [100]. Merten *et al. *[102] reported a negligible incidence of CIN in subjects treated with a sodium bicarbonate infusion at 3 ml/kg/h before contrast followed by 1 ml/kg/h after contrast. These studies suggest that fluid administration regimens have a large impact on CIN risk. It is worth noting that all four highly beneficial studies in cluster 2 of our meta-analysis [10,11,14,25] employed protocols specifying half-normal saline infusions at 1 ml/kg/h.

Changes in serum creatinine levels have invariably been used to diagnose CIN in trials of NAC. However, serum creatinine is a poor surrogate marker for glomerular filtration rate (GFR) because creatinine is influenced by diet, endogenous production, renal filtration, secretion and reabsorption [103,104]. Contrast agents themselves may decrease creatinine secretion and thereby raise serum creatinine levels, independently of changes in GFR [105]. Conversely, NAC in the absence of contrast has been shown to decrease serum creatinine levels in normal volunteers [106] and patients [66]. Hoffmann *et al. *[106] detected significant NAC-induced decreases in serum creatinine that were not associated with similar changes in cystatin C. As cystatin C is not secreted by renal tubule cells it may be a more accurate indicator of GFR [107,108]. Interestingly, in our meta-analysis, three out of the four cluster 2 studies [10,11,14] and one cluster 1 study [17], shown by sensitivity analysis to make a relatively large contribution to heterogeneity, all reported substantial NAC-induced decreases in serum creatinine. This response to NAC may be a drug effect independent of changes in GFR.

The four highly beneficial studies (cluster 2) represent only 11% of patients in our meta-analysis. These trials were significantly different from cluster 1 studies in that they had early publication dates, were small in size and of low quality. Furthermore, cluster 2 studies uniformly employed an inferior hydration regimen that may have exaggerated any effects of NAC treatment. Cluster 2 studies were characterized by relatively large serum creatinine increases in control patients and similarly large creatinine decreases in NAC-treated patients.

A power analysis of cluster 1 studies indicated that 32 200 patients would be needed in a single PRCT to have an 80% chance of detecting benefit using definitions of CIN based on serum creatinine. Importantly, dialysis use was not decreased by NAC treatment across the 2746 patients in our meta-analysis. The large PRCT just proposed would have a moderate likelihood of demonstrating harm as measured by the more rigorous clinical endpoint of dialysis. Based on this investigation, low-dose oral NAC has not been shown to prevent CIN and should not be routinely recommended.

Eight of the nine new trials published since we closed our meta-analysis [58-63,70,71] were found to group with cluster 1 and support our overall findings. One of the trials was an outlier and not only reported significant reductions in CIN rates, but also decreases in dialysis use and mortality [64]. In this study, very ill patients with acute myocardial infarctions were treated with intravenous NAC boluses during angioplasty [64]. As noted by the authors, these single-center results require confirmation. As survival improved in their trial, Marenzi *et al*. speculated about possible benefits of NAC beyond the simple prevention of CIN [64]. Alternatively, the relatively high mortality in control subjects might also be explained by hidden imbalances created during randomization. In contrast to this highly beneficial trial, other studies in high-risk patients undergoing coronary bypass [109] or abdominal aortic surgery [110] did not find that intravenous NAC reduced the incidence of postoperative renal dysfunction or mortality.

## Conclusion

Our meta-analysis does not support the use of NAC for reducing rates of acute kidney injury due to intravascular iodinated contrast. In several overly influential trials showing large beneficial effects, NAC decreased serum creatinine levels, suggesting possible drug effects independent of true changes in GFR. Dialysis use across all studies occurred infrequently, but did not indicate that NAC was efficacious. Future clinical trials of therapies to prevent CIN should incorporate primary endpoints other than change in creatinine.

## Competing interests

The author(s) declare that they have no competing interests.

## Authors' contributions

RLD, KJN and DAG conceived of the study protocol. PCS, KJN, SJK, DAG and RLD were responsible for collecting and assembling source data. KJN, SJK and SB provided statistical expertise. RLD, SJK, SB, DAG, CN and KJN were responsible for analyzing and interpreting data. All authors read and approved the final manuscript.

## Pre-publication history

The pre-publication history for this paper can be accessed here:


